# Albright’s Hereditary Osteodystrophy: A Rare Genetic Disorder Diagnosed on Standard Radiography

**DOI:** 10.5334/jbsr.3690

**Published:** 2024-08-27

**Authors:** Catherine Dessard, Jacques Malghem, Lokmane Taihi

**Affiliations:** 1 Department of Medical Imaging, University Hospital of Liège, avenue de l’Hôpital 1, 4000 Liège, Belgium; Department of Medical Imaging (Musculoskelettal Imaging Unit), Saint-Luc University Clinics, avenue Hippocrate 10, 1200 Bruxelles, Belgium; 2 Department of Medical Imaging (Musculoskelettal Imaging Unit), Saint-Luc University Clinics, avenue Hippocrate 10, 1200 Bruxelles, Belgium; 3 Department of Medical Imaging (Musculoskelettal Imaging Unit), Saint-Luc University Clinics, avenue Hippocrate 10, 1200 Bruxelles, Belgium

**Keywords:** Brachyphalangies, brachymetacarpies, Albright’s hereditary osteodystrophy, soft tissue calcifications, pseudohypoparathyroidism

## Abstract

*Teaching point:* Some genetic syndromes have characteristic features that allow for their diagnosis to be made based on radiological findings.

## Case History

**Figure 1:**
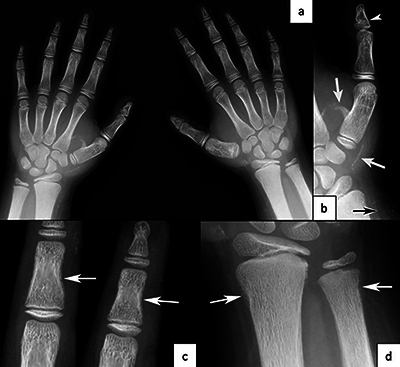
**a)** Hands radiography for bone age assessment. Slight brachymetacarpy of the 3rd, 4th and 5th metacarpals and brachyphalangy (P2R1/P2R5). **b)** Small distal phalanx of both thumbs due to premature fusion of growth plate (arrow head), pseudo-exostosis and linear ossification (arrows). **c)** and **d)** Resorption of subperiosteal bone (arrows).

**Figure 2:**
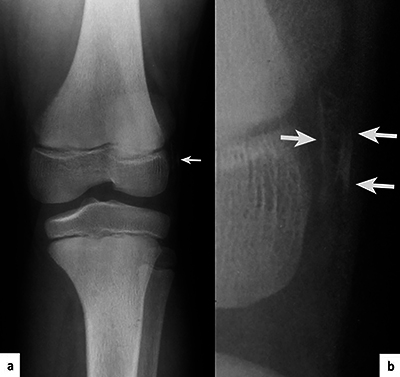
Thin linear ossifications of the soft tissues (arrows) on the lateral side of the lateral condyle, parallel to the skin, visible on the knee X-ray **(a)** and the magnified view **(b)**.

A nine-year-old child was referred for a hands X-ray examination for short stature investigation (Figure 1). A previous knee X-ray was available (Figure 2), and a foot X-ray was performed (Figure 2).

**Figure 3:**
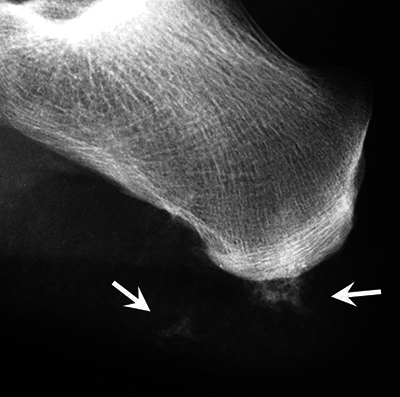
Calcifications/ossifications of the soft tissues next to the posterior tuberosity of the calcaneus.

These X-ray images reveal several semiological elements:

- Brachytelephalangy of the thumbs (Figure 1b, arrowhead).
- Brachymetacarpies and other brachyphalangies of different degrees (Figure 1a).
- Calcifications/ossifications in the soft tissues and pseudo-exostosis (Figure 1b, Figure 2, Figure 3, arrows).
- Some foci of subperiosteal resorption (Figure 1c, Figure 1d, arrows).

These elements are indicative of Albright’s hereditary osteodystrophy (pseudohypoparathyroidism) associated with secondary hyperparathyroidism. The radiological diagnosis was confirmed by genetic analysis.

## Comments

Albright’s hereditary osteodystrophy is a genetic disorder with its expression depending on the mode of inheritance and the type of guanine nucleotide binding protein alpha–stimulating activity polypeptide (GNAS) gene mutation. This mutation may induce resistance to parathyroid hormone.

It is associated with morphological anomalies such as short stature, round face, obesity, skeletal involvement (brachymetacarpy, brachymetatarsy, brachyphalangy, exostoses, …), calcifications/ossifications of the soft tissues, and calcifications of the basal ganglia of the brain. Signs of secondary hyperparathyroidism (subperiosteal resorption) may be observed in children.

There are two types, with similar clinical manifestation. Pseudohypoparathyroidism (PHP), or type 1, is associated with hypocalcemia and hyperphosphatemia and with the body’s not responding to parathyroid hormone. Pseudopseudohypoparathyroidism (PPHP), or type 2, is characterized by normocalcemia and a normal response to parathyroid hormone.

The range of skeletal anomalies is widely variable. However, the distal phalanx of the thumb is the most commonly shortened bone. This sign is highly suggestive of PHP/PPHP [1].

Brachymetacarpy of the 4th finger is most frequently observed. This non-specific sign can be found in other diseases such as Turner syndrome. However, when this sign is associated with brachytelephalangy of the thumb, it becomes a more specific sign [1].

The condition is often asymmetrical.

## Conclusion

This rare condition can be diagnosed based on standard X-rays.
